# Lassar’s Paste for Eczema

**Published:** 1898-09

**Authors:** 


LASSAR'S PASTE FOR ECZEMA. ℞.—Pulv. acidi salicylici, grs. lx; pulv. amyli et zinci oxidi, āā ʒiij et grs. xx; petrolatum, ʒvj et grs. 40. M. This paste is extremely useful in the early and moist stages of eczema..

A STYPTIC. Antipyrine in powder applied to a bleeding surface acts as a prompt styptic.

